# Medical students’ learning experience and participation in communities of practice at municipal emergency care units in the primary health care system: a qualitative study

**DOI:** 10.1186/s12909-022-03492-7

**Published:** 2022-06-02

**Authors:** Solveig Giske, Marit Kvangarsnes, Bodil J. Landstad, Torstein Hole, Berit Misund Dahl

**Affiliations:** 1 Department of Health Sciences in Ålesund, Faculty of Medicine and Health Sciences, Norwegian University of Science and Technology (NTNU), Ålesund, Norway; 2 Department of Health Sciences, Mid Sweden University, Östersund, Sweden; 3 Medical Department, Ålesund Hospital, Møre and Romsdal Hospital Trust, Ålesund, Norway

**Keywords:** Undergraduate medical education, Medical students, Learning, Primary health care, Emergency medicine, Interprofessional education, Interprofessional collaboration, Community of practice

## Abstract

**Background:**

Medical education has been criticised for not adapting to changes in society, health care and technology. Internationally, it is necessary to strengthen primary health care services to accommodate the growing number of patients. In Norway, emergency care patients are increasingly treated in municipal emergency care units in the primary health care system. This study explores medical students’ learning experience and how they participated in communities of practice at two municipal emergency care units in the primary health care system.

**Methods:**

In this qualitative study, we collected data from March to May 2019 using semi-structured individual interviews and systematic observations of six ninth-semester medical students undergoing two-week clerkships at municipal emergency care units. The interview transcripts were thematically analysed with a social constructivist approach. A total of 102 systematic observations were used to triangulate the findings from the thematic analysis.

**Results:**

Three themes illuminated what the medical students learned and how they participated in communities of practice: (i) They took responsibility for emergency care patients while participating in the physicians’ community of practice and thus received intensive training in the role of a physician. (ii) They learned the physician’s role in interprofessional collaboration. Collaborating with nursing students and nurses led to training in clinical procedures and insight into the nurses’ role, work tasks, and community of practice. (iii) They gained in-depth knowledge through shared reflections when time was allocated for that purpose. Ethical and medical topics were elucidated from an interprofessional perspective when nursing students, nurses, and physicians participated.

**Conclusions:**

Our findings suggest that this was a form of clerkship in which medical students learned the physician’s role by taking responsibility for emergency care patients and participating in multiple work tasks and clinical procedures associated with physicians’ and nurses’ communities of practice. Participating in an interprofessional community of practice for professional reflections contributed to in-depth knowledge of ethical and medical topics from the medical and nursing perspectives.

**Supplementary Information:**

The online version contains supplementary material available at 10.1186/s12909-022-03492-7.

## Background

Clerkships for medical students are designed to gradually improve their abilities in assessing and treating patients, working in teams, and undertaking the role and professional identity of a physician [1]. Over the past century, most clerkships have occurred in community and teaching hospitals. However, in recent decades, an increasing amount of clinical training has taken place in primary health care settings [2, 3]. The adjustment of medical clerkships is a response to criticism of medical education for not adapting to changes in society, the health care system, and technology [1, 2, 4]. These changes demand a more sustainable and collaborative health care system and place pressure on medical education to adapt [4, 5]. For many developed countries, such as Norway, the UK, the Netherlands, and Australia, one measure has been to strengthen primary health care [6–9].

In Norway, municipalities provide inpatient emergency care for patients with somatic, mental, or substance use problems [6, 10]; this care is provided in settings known as municipal emergency care units. Before admission, a family or emergency room physician must judge that the severity of the patient’s condition is manageable within the unit’s infrastructure and expertise [10]. Typical conditions include exacerbation of known heart failure and chronic obstructive pulmonary disease, pneumonia, urinary tract infections, fractures, concussions, mental health, and substance use problems [10]. A pilot study explored the use of a municipal emergency care unit as a learning arena for medical students [11]. The findings showed that medical students received training in various practical duties of a physician, especially with elderly patients; learned to collaborate interprofessionally with nurses; and gained insight into the importance of collaboration with other municipal health care services and secondary health care and how it takes place [11]. The present study explores what medical students learned and how they participated in communities of practice at two municipal emergency care units in the primary health care system.

Instruction in emergency medicine is considered a vital part of undergraduate medical education [12–14] and occurs in emergency departments at teaching and community hospitals [15–17]. Despite detecting little evidence of such clerkships within primary health care, Grove et al. [18] found that primary care settings may, during off hours, provide acute care training for medical students. Medical students undergoing emergency medical clerkships have reported increased confidence in assessing, diagnosing and managing acutely ill patients and performing clinical procedures [19]. However, patient encounters depend on the setting, quantity and severity of patients. Medical students have reported that they primarily met patients with chest pain, abdominal pain and respiratory distress. Cardiac arrest, gastrointestinal bleeding, and shock were the least frequently encountered patient conditions or diagnoses [20, 21]. The time spent in an emergency medicine clerkship also varies, but both longer and shorter clerkships are considered to provide valuable curricular core competencies [13, 19].

The combination of physician shortages, an increased number of patients with complex health care needs and fragmented health care systems creates a need to implement interprofessional education of medical students [22]. According to the World Health Organisation (WHO), interprofessional education occurs when students from two or more professions learn from, with, and about each other to improve collaboration and health outcomes [22]. Governmental, clinical and educational support is found to affect the development and sustainability of interprofessional education [23]. In addition, supervisors promoting safe learning environments and facilitating interactions between students from various professions are vital prerequisites for interprofessional education, as is the students’ desire to participate [23]. Interprofessional education in clinical settings for medical, nursing, and allied healthcare students has been found to increase their knowledge of each other’s professional roles and collaborative skills [24–26] and view patients from different professional or holistic perspectives [27–29]. In addition to patient collaboration, joint discussions seem to positively influence interprofessional education, contributing to increased insight into other professions’ perspectives and knowledge [30–32].

Learning in primary health care settings is valued by medical students [33, 34] and provides academic results comparable to those of peers undergoing traditional hospital block rotations [33–35]. Medical students have reported meeting patients with acute, chronic, and common health problems [33, 36, 37] as well as multimorbidity [36, 38]. Working with patients provided learning outcomes related to practising communication, physical examination, clinical decision making [33, 36], and assessing patients in a holistic perspective related to comorbidity [36] and the psychosocial and environmental factors that can affect health [33, 37]. Other learning outcomes included preventive medicine [37, 39], medication management [37, 38], record keeping, writing referrals, and developing management plans [36–38]. Medical students have also reported learning to collaborate interprofessionally with nurses and other health care staff [37, 38] and gain insight into how primary health care is organised [36–38]. In addition to the diversity of learning outcomes, undertaking primary care clerkships and experiencing a positive learning environment may motivate medical students to choose primary health care as their future career path [33, 34, 40].

This study used Wenger’s social theory of learning as the theoretical framework [41]. Wenger proposes that learning occurs in social interactions in a community of practice (CoP). A CoP can be defined as a group of people with shared interests who learn how to perform better through social interactions [41]. A CoP consists of three dimensions: (i) mutual engagement, (ii) joint enterprise, and (iii) shared repertoire. Mutual engagement involves the members’ interactions, interest, and commitment to the common practice in the CoP. Joint enterprise refers to the unified goal of the practice that is negotiated among the members. Shared repertoire consists of the norms, routines, actions, words, and concepts that the community uses to negotiate meaning and govern how the members interact [41].

Central concepts in Wenger’s theory are legitimate peripheral participation and trajectories [41]. Legitimate peripheral participation, first described by Lave and Wenger [42], involves the process of being included in a CoP. Trajectories describe forms of participation, and Wenger refers to them as peripheral, inbound, insider, boundary, and outbound [41]. Peripheral trajectories do not lead to full access to a CoP but can provide a form of access. Individuals participate with the goal of becoming full members through inbound trajectories; this process is central for clerkship students. Insider trajectories refer to the continuous development of professional identity even after becoming a full member. Boundary trajectories involve connections linking the community to other relevant CoPs, for example, through interprofessional teamwork. Outbound trajectories lead out of a CoP, but such a path can contribute to identity development and participation in other CoPs. To become a member of a CoP, the student must be legitimised. Which trajectory the student follows affects the development of professional identity. Professional identity is continually forming and involves a gradual change in how the student views himself or herself and how others view the student [41]. CoP theory is further developed through the argument that learning takes place not only in an individual CoP but also in a “landscape of practice” where learning may occur within several CoPs [43].

Previous studies have investigated medical students learning emergency medicine and interprofessional collaboration in primary health care settings, reporting on their learning outcomes. However, little is known about what medical students learn at municipal emergency care units in the primary health care system. This study explores what medical students learn and how they participate in CoPs at municipal emergency care units in the primary health care system. Findings may provide insight into necessary competence for future work in this arena and may influence clerkship development and medical education. Because this learning occurs in a medical context, we believe that findings can be transferred to other medical or similar clerkship arenas. The following research question guided this study:

What do medical students learn and how do they participate in CoPs at municipal emergency care units in the primary health care system?

## Methods

### Design

This study had a qualitative design with a social constructivist approach [44–46]. Social constructivists assume a relativist ontology, meaning there are multiple realities with no objective truth [44]. Knowledge is socially constructed among individuals’ shared experiences within a setting [46]. Epistemologically, knowledge is subjective and based on interactions between the researcher and the study participants [44, 45]. Thus, we used individual interviews and systematic observations to collect data [44, 47].

### Participants

Six medical students from a Norwegian university were chosen by purposive sampling [48, 49] based on their experience with clerkships at a municipal emergency care unit. The number of students was predetermined due to professional and structural resources at the clerkship sites. Before the municipal emergency care clerkship, 19 medical students were eligible to participate in this study. A clerkship coordinator contacted the medical students by email or phone and asked them to participate. The first 6 medical students who agreed to participate were accepted. The medical students consisted of four females and two males, aged 24 to 28 years (mean age, 26 years), and none withdrew from the study. The inclusion criteria for participation included being in the ninth semester (out of twelve), speaking Norwegian, attending a clerkship in internal medicine in secondary health care services, and having an interest in participating in this study.

### Setting

The study setting took place at two municipal emergency care units in two cities under the Central Norway Regional Health Authority. The medical students were organised in a joint clerkship with fourth-semester (out of six) student nurses to collaborate on patients. Two weeks of their clinical clerkship in medicine was replaced with 2 weeks at municipal emergency care units, focusing on emergency care patients. A daily plan adapted to the structure of the municipal emergency care units was developed. The plan contained a step-by-step overview of the day, involving a morning meeting, preparations, pre-rounds, physician rounds, clinical work including documentation, and an afternoon meeting where professional reflections took place. The steps contained instructions for work tasks, facilitated collaboration with student nurses, and indicated when the observations were to occur.

### Data collection

Individual semi-structured interviews and systematic observations were conducted between March and May 2019. The interviews, which lasted 60 to 94 minutes, were audio recorded and later transcribed verbatim by the first author, SG [49, 50]. Before the interviews were conducted, an interview guide was developed based on individual and sociocultural perspectives on learning [41, 51]. The questions covered topics concerning what the medical students learned and how they participated in this particular clerkship (Table 1). The systematic observations were based on the same areas that the interview guide covered (Table 1). “Systematic observations” means that the areas to be observed and how they are registered on an observation tool are predetermined [47]. A total of 102 systematic observations were performed by SG, BMD, MK, and BJL. The observers noted in free text how the medical students participated, and they used a pre-developed observation tool to register with whom the medical students interacted and the setting of the learning situation associated with the plan of the day. The medical students were observed 2 days per week during the daytime for 2 weeks, and each observation lasted approximately 15 minutes.

**Table 1:** Interview guide and situations where systematic observations took place

**Interview guide**	
• How did you experience the clerkship at the municipal emergency care unit?	
• How did you experience participating in the community of practice?	
• How did you experience interacting and collaborating on work tasks?	
• How did you participate in work tasks?	
• What have been your learning outcomes?	
• How did you experience the balance between support and challenges?	
• How did you receive feedback?	
• How did you experience learning and participating at the afternoon reflection meetings?	
**Systematic observations**	
• Morning meeting	
• Preparations	
• Pre-rounds	
• Physician rounds	
• Clinical work	
• Afternoon reflection meeting	

### Data analysis

The interview transcripts were analysed using a social constructivist approach to search for social learning situations occurring when the medical students interacted and collaborated with physicians, student nurses, nurses, and patients using a six-step thematic analysis guided by the methodology of Braun and Clark: 1) familiarization with the data, 2) generating initial codes, 3) searching for themes, 4) reviewing themes, 5) defining and naming themes, and 6) writing the report [50]. As the researchers followed the steps in a non-linear process and moved within and between the interview transcripts, patterns in the data were identified, analysed, and sorted into themes [50]. NVivo software version 12.0 was used to assist with coding the transcripts and developing a thematic map [52, 53]. Throughout the analytical process, all questions and disagreements about coding and abstractions were openly and carefully discussed among the mentioned authors in the respective steps until a consensus was reached.

First, SG, BMD, and MK individually and thoroughly reviewed the interview transcripts while noting and discussing initial ideas for coding where the social learning situations took place. Second, SG sorted all relevant data that formed patterns into initial codes. In the third step, SG applied the research question and theoretical framework [41] to guide the sorting of the initial codes into potential sub-themes and themes, forming an initial thematic map. BMD and MK contributed by carefully reviewing the second and third steps in the analytical process. The fourth step involved all authors who reviewed the potential themes and quotations. Fifth, the authors abstracted, refined, and named the themes and finalised the thematic map. Finally, SG wrote the report with iterative review from the co-authors to strengthen the presentation and validity of the findings. In the last step, SG sorted the observational data into different learning situations. The authors collecting the systematic observations triangulated these data to substantiate the findings in the thematic analysis and discussed them to ensure that they were adequately accounted for in the report. Table 2 presents quotations and observational data to illustrate the development of the themes.

**Table 2:** Development of themes

Quotes and observations	Sub-theme	Theme
**Quote** “[…] learn a little about the interactions and thinking behind the [medication] changes […] And then, maybe I say something that I have learned. And then, it is discussed.” (MS5)	Assessing patients during pre-rounds	Taking responsibility for emergency care patients
**Observation** The medical students and physicians sat together at a table with charts or computers available. Most physicians facilitated active participation by engaging the students in the discussions about the patients, asking questions, and encouraging them to assess the patients’ condition.		
**Quote** “[…] something that has been very nice is the time you get with the physician, where you make suggestions, or you have examined a patient, and then you can talk about it and what to do next” (MS1)	Developing independence when interacting with patients	
**Observation** We observed the medical students as they performed physician rounds. When a physician accompanied them, the physician allowed the student to take the lead by keeping in the background. However, the physician sometimes interrupted with input. When no physician was present, we observed how the students approached the patients naturally.		
**Quote** “I am very aware that one should write good discharge notes when I now suddenly sit on the other side and will receive them [the patients]. […] a lot of the treatment here is based on what is written in the discharge note from the hospital.” (MS2)	Keeping proper documentation	
**Quote** “There are some who are very good nurses […], I feel like […] standing there with hat in hand. […] you get away with that when it is a student nurse” (MS2)	Learning to lead when collaborating with student nurses	Learning the physician’s role in interprofessional collaboration
**Observation** There were several situations where medical students interacted and collaborated with nursing students. For example, medical students met nursing students at morning meetings to plan the day and decide which patients to collaborate on. Most medical students took on the physician’s role while collaborating with nursing students on assigned patients in pre-rounds and physician rounds. During the day, short meetings in the hallway took place, where the medical and nursing students discussed patients and further work tasks.		
**Quote** “It matters that you get to see more of what all the professions do. […] what the patients go through from A to Z during the admission period” (MS6)	Learning about the nurse’s role	
**Observation** The nursing students and nurses contacted the medical students when clinical procedures were available and provided the necessary support to complete the clinical procedures.		
**Quote** “Talking aloud about difficult things is useful in itself. […] hearing that other people may think the same and may see the same challenges and think that things may be a little difficult or, yes. That one is not alone about things.” (MS4)	Developing ethical competencies	In-depth knowledge through shared reflections
**Observation** The medical students engaged in ethical reflections with the nursing students, contributed actively and showed interest in their perspectives.		
**Quote** “That we could ask them. […] Don’t you who have been doing this for ten years any suggestions? And then they sometimes have a little, some nice things that I will take with me further...” (MS3)	Developing content understanding	
**Observation** The physicians and nurses became involved in medical topics covering various patient situations and diagnoses such as multimorbidity, diabetes, and heart failure. Observations also showed that when the physicians and nurses asked the medical students questions about the addressed topics at hand, this pushed the reflections deeper and facilitated higher-order thinking.		

### Reflexivity

Reflexivity is cardinal for qualitative researchers to identify personal biases and background that may shape the study’s interpretation of data and direction [54]. The authors are researchers and employees at a university with health education and a hospital. Our preunderstanding of the field was carefully considered through critical reflection and discussion to avoid a biased interpretative process and direction of the study. To promote quality, we used the Standards for Reporting Qualitative Research (SRQR) checklist [55] (see Additional file 1).

### Ethics

The medical students were informed about the study both orally and in writing. They were informed that participation was voluntary, that they could withdraw at any time without stating a reason or facing consequences in their studies, and that the data gathered would be handled confidentially. An application was sent to The Norwegian Centre for Research Data (NSD, reference number 602973), which approved the study in compliance with privacy legislation.

## Results

Three themes were identified that illuminated what the medical students learned and how they participated in CoPs at two municipal emergency care units in the primary health care system: (i) they took responsibility for emergency care patients; (ii) they learned the physician’s role in interprofessional collaboration; and (iii) they gained in-depth knowledge through shared reflections. In the triangulation of the observational data, we found that they mainly corresponded well with what the medical students reported. We did not experience any particular negative observations.

### Taking responsibility for emergency care patients

The medical students described participating in the daily tasks of a physician, such as pre-rounds, physician rounds, examination, follow-up, documentation for medical records, and training in clinical procedures. Their continuous presence in the unit was important for their familiarization with the physicians and routines. While initially mostly observing, the students reported gradually gaining access to and taking on responsibility for intensive training in work tasks and patient care. This gradual progression was explained by their familiarization with their potential work tasks and by the physicians’ need to assess their competence before entrusting them with responsibility. However, while some medical students reported that they had wanted the physicians to be more available in the beginning, there was also a desire to be given more responsibility. Being assigned responsibility was reported to be positive for learning independence and increased their self-confidence regarding their development as physicians.

Participation in pre-rounds was expressed as an arena for assessing the patients’ conditions, treatment, medications, bloodwork and planning further follow-up in primary health care. The patients were reported to be discussed from a holistic perspective, and discussion was specifically related to the reason for admission. While learning about the patients and how the pre-rounds took place by observing the interactions and discussions between the physicians and nurses, some students reported that they particularly learned when actively participating in the discussions, by asking questions, and when the physicians spent time explaining:

*“[…] learn a little about the interactions and thinking behind the [medication] changes […] And then, maybe I say something that I have learned. And then, it is discussed.” (MS5)*

We observed how the medical students and physicians sat together at a table with charts or computers available. Most physicians facilitated active participation by engaging the students in the discussions about the patients, asking questions, and encouraging them to assess the patients’ condition. Some students participated more actively than others.

Patient interactions took place during physician rounds, follow-up conversations throughout the day, examinations, and clinical procedures. Some students stated that they were accompanied by a physician on the physician rounds, while others went without a physician. However, both types of experience were reported to provide learning outcomes related to communicating with patients. The students who were accompanied by a physician expressed that they either observed when the physician talked to the patients or were assigned to do the talking. In the latter situation, one student reported the dialogue with the patients’ as more realistic when allowed to talk to the patients without the physician interrupting, as this could lead to losing track of the conversation. However, it was appreciated if the physician joined at the end and supported when needed. We observed the medical students as they performed physician rounds. When a physician accompanied them, the physician allowed the student to take the lead by keeping in the background. However, the physician sometimes interrupted with input. When no physician was present, we observed how the students approached the patients naturally.

The medical students compared this clerkship with the one at the hospital, and some highlighted that the municipal emergency care unit had a slower pace of work. Being in the unit for several days made it easier for them to make contact, establish professional relationships, and follow up patients through their pathway. Most patients were reported to be elderly people with multimorbidity and conditions such as heart failure, pneumonia, urinary tract infection, fractures, chronic pain, hearing and vision impairment, and cognitive impairment related to dementia and delirium. Some students also encountered terminal and deceased patients. The students reported that they were assigned new emergency care patients and followed up already admitted patients with whom they received intensive training in communication and examination. Some reported particularly challenging communication situations with patients with chronic pain, impaired hearing, or cognitive impairment or those who were critically ill or dying. The decreased time pressure compared to the hospital clerkship created opportunities to have in-depth conversations with patients about their condition and how they looked upon the future. The students reported that they examined patients who had already been assessed and diagnosed on arrival, which allowed them to shorten the assessments and tailor each one to the particular patient’s condition. However, while some students expressed missing the opportunity to examine undifferentiated patients, some students reported being involved in examining the patients for diagnosis when the initial diagnosis was unclear or wrong or when new medical problems appeared. They explained that they conducted the examinations by assessing the patients’ symptoms against bloodwork and X-rays and planning additional examinations to diagnose the problem.

The medical students appreciated spending time with the patients and described them as friendly and patient. We observed that the patients were interested in the medical students, agreeing and some even requesting to be examined. The students expressed that they valued when the physicians facilitated clinical procedures on patients, such as listening to heart murmurs, evaluating cognitive function, performing hand-held ultrasound (Vscan) of the lungs, inserting permanent bladder catheters, drawing arterial blood, and performing skin biopsies. When the students worked with patients, they reported interacting with the physicians as needed by asking questions or requesting confirmation that their actions were correct. How the physicians provided feedback affected the medical students’ perceived development of independence. When the physicians awaited their feedback and gave the students time to think aloud and suggest actions, this contributed to the development of independence:

“*[…] something that has been very nice is the time you get with the physician, where you make suggestions, or you have examined a patient, and then you can talk about it and what to do next.” (MS1)*

In contrast, when the physicians on beforehand revealed their thinking and further actions, this was expressed to inhibit the development of independent thinking skills.

The medical students reported receiving intensive training on documenting information in the electronic medical records. The documentation system was new to the students, and most reported that they initially spent a considerable amount of time familiarizing themselves with its use. Although they mainly wrote admission and discharge notes, some also mentioned writing progress notes and medication administration records. Learning how the admission and discharge notes were written at the municipal emergency care unit and the significance of thorough documentation was valued:

*“I am very aware that one should write good discharge notes when I now suddenly sit on the other side and will receive them [the patients]. […] a lot of the treatment here is based on what is written in the discharge note from the hospital.” (MS2)*

Most physicians were reported to provide feedback on the medical students’ writing, which was appreciated. Although mostly positive, the physicians’ feedback also included constructive criticism, for example, instructing the students to chart more efficiently and to include further follow-up in the discharge note.

### Learning the physician’s role in interprofessional collaboration

The medical students reported collaborating interprofessionally with nursing students on patient care in learning activities such as pre-rounds, physician rounds, follow-up, and clinical procedures. Collaborating with nursing students was reported to increase independence, decision making, and self-confidence related to learning the physician’s role. There was variation in how much and on which work tasks the medical and nursing students collaborated. For example, during pre-rounds, some medical students described participating by observing and providing input or discussing patients. However, in accordance with our observations, most participated by practising having pre-rounds on joint patients in collaboration with a nursing student. Medical students who mainly observed pre-rounds expressed a wish to have had a more participatory role in the pre-rounds and that they did not learn as much from just observing and providing input.

Some medical students expressed that collaborating on learning activities such as pre-rounds and following up patients involved a new experience of giving messages to the nursing students about what to do with the patients. While looking at the nursing students as learners on the same footing as themselves, the medical students found it challenging to speak directly as physicians and thus conveyed messages in a more reluctant way. After becoming more acquainted, some medical students stated that the nursing students started asking them professional questions about the patients and their condition, making them aware of their knowledge. While assessing and discussing the patients’ conditions and treatment plans with the nursing students, the medical students used both the medical perspective and the nursing students’ reports on the patients’ vital signs and current condition. Some medical students reported that assessing patients with nursing students was beneficial because it contributed to more realistically distributed roles and provided the opportunity to lead. One example involved a patient whose condition was worsening. In collaboration with a nursing student, the medical student reported assessing the patient’s condition and considering a hospital transfer. In contrast, working with an experienced nurse could make medical students less inclined to speak:

*“There are some who are very good nurses […], I feel like […] standing there with hat in hand. […] you get away with that when it is a student nurse.” (MS2)*

We observed several situations where medical students interacted and collaborated with nursing students. For example, medical students met nursing students at morning meetings to plan the day and decide which patients to collaborate on. The observations showed that most medical students took on the physician’s role while collaborating with nursing students on assigned patients in pre-rounds and physician rounds. During the day, short meetings in the hallway took place, where the medical and nursing students discussed patients and further work tasks.

Collaborating with nursing students on patient care was described by the medical students to provide insight into the nurses’ role and how they thought and worked; the medical students considered this to be convenient knowledge for future collaboration. For example, the medical students expressed that it was useful to know what information the nursing students were interested in obtaining from the patients during admission and their perspectives in the care and treatment of the patients:

*“It matters that you get to see more of what all the professions do. […] what the patients go through from A to Z during the admission period.” (MS6)*

The medical students valued collaboration with nursing students and nurses in practising available clinical procedures related to learning outcomes, and they selected procedures they found most useful. While the nursing students tutored them in drawing blood, the nurses instructed or supported the medical students in drawing and analysing arterial blood, inserting peripheral intravenous needles, setting up and administering intravenous infusions, setting up pain pumps, inserting permanent urinary catheters, and assessing wounds. The nursing students and nurses were described as showing interest in the medical students, making them feel welcomed and supported. We observed that nursing students and nurses contacted the medical students when clinical procedures were available and provided the necessary support to complete the clinical procedures.

### In-depth knowledge through shared reflections

The medical students described participating with the nursing students at afternoon reflection meetings, finding relevant topics related to this clerkship. The topics covered various medical and ethical aspects, how municipal emergency care is organised, and experiences with collaborating on patients. We observed that the physicians and nurses attended the meetings whenever possible.

Ethical topics were expressed to be particularly valuable for reflections with the nursing students that contributed to developing ethical competencies. The nursing students added novel knowledge to the reflections; elucidated the nursing perspective; and provided insight into the nurses’ role, how they think, and what they consider important for patient care. Discussing ethical issues drew medical students’ attention to challenging topics such as communicating with patients with anxiety or cognitive impairment, communicating resuscitation plans to patients with short life expectancy and their relatives, and dilemmas regarding withholding sweet cakes from elderly diabetic patients:

*“Talking aloud about difficult things is useful in itself. […] hearing that other people may think the same and may see the same challenges and think that things may be a little difficult or, yes. That one is not alone about things.” (MS4)*

We observed how the medical students engaged in ethical reflections with the nursing students, contributed actively, and showed interest in their perspectives. However, regarding medical topics, some medical students reported fewer mutual discussions, which was explained by their different professional backgrounds. It was expressed to be challenging to find medical topics suitable for both professions, and some suggested that a list of medical topics provided could have been useful.

The medical students expressed that when the physicians and nurses participated in the reflection meetings, they contributed to an increased understanding of content concerning the topics discussed. For example, physicians’ and nurses’ input on ethical reflections provided novel and in-depth knowledge on how to approach challenging situations:

*“That we could ask them. [...] Don’t you who have been doing this for ten years any suggestions? And then they sometimes have a little, some nice things that I will take with me further...” (MS3)*

However, the medical students also expressed appreciation when the physicians elaborated on medical topics such as treatment with warfarin, increasing and clarifying the students’ knowledge related to the medical profession. The medical students expressed—and we observed—how the physicians and nurses became involved in medical topics covering various patient situations and diagnoses such as multimorbidity, diabetes, and heart failure. Observations also showed that when the physicians and nurses asked the medical students questions about the addressed topics at hand, this pushed the reflections deeper and facilitated higher-order thinking.

## Discussion

This study aimed to explore what medical students reported learning and how they participated in communities of practice (CoPs) at municipal emergency care units in the primary health care system. We found that this clerkship was complex and provided multiple learning opportunities. Our main findings showed that the medical students took responsibility for emergency care patients by gradually participating in the physician’s CoP and thus received intensive training in work tasks and clinical procedures relevant to their profession. Furthermore, the medical students learned to collaborate interprofessionally with nursing students by collaborating on patients and received training in clinical procedures with nursing students and nurses, which contributed to insight into the nurses’ CoP and how nurses work in close partnership with physicians in patient care. In addition, the medical students developed in-depth knowledge about ethical and professional topics and the nursing profession through shared reflections with student nurses, nurses, and physicians. Overall, we found that the municipal emergency care clerkship provided very positive learning experiences, and only a few suggestions for improvements were reported from the medical students. This was in accordance with our observations.

The medical students were given responsibility for emergency care patients. Although a physician assesses emergency care patients at municipal emergency care units before admission [10], we found that some students encountered patients who needed further examinations or whose medical condition worsened. Manthey et al. [14] noted that medical students should learn to assess acutely ill and undifferentiated patients. However, Shaban et al. [21] found that most medical students do not encounter all recommended patient presentations in emergency medicine clerkships. Because emergency medicine is usually learned in hospital settings, municipal emergency care units cannot be directly compared. However, the trend is that patients with exacerbation of one or more chronic conditions are increasingly treated in primary care [4, 5]. In addition, Grove et al. [18] showed that, although out of hours, primary care can offer acute patient presentations. Based on our findings and earlier research, we believe that municipal emergency care units may provide valuable learning situations for practising emergency medicine on relevant patients in primary health care.

The medical students expressed appreciation for the opportunity to gradually work more independently with patients and take on the tasks of a physician, consistent with Wenger’s concepts of legitimate peripheral participation in a CoP and movement along an inbound trajectory, which are vital for developing the physician’s role and professional identity [41]. This finding is supported by Davies et al. [40] and Eggleton et al. [56], who reported that gradually gaining autonomy and taking responsibility for patients positively influenced students’ development into physicians. Thus, gaining responsibility seems to lead students on inbound learning trajectories and affect their degree of perceived legitimacy in the physicians’ CoP [41]. The opportunity to participate actively indicated mutual engagement between the medical students and the physicians, with the goal of enabling the student to learn the physician’s role [1, 41]. The students’ interactions with the physicians helped build a shared understanding or joint enterprise for the physician’s purpose of patient treatment in the unit. In the learning process, the medical students learned the routines, clinical work, and professional communication expected of a physician, thus building a shared repertoire with the physicians [41]. The medical students in our study valued feedback on how they reasoned on patient assessment; this finding was consistent with a report by Salminen et al. [57], who found that feedback supporting the development of independence is considered important by medical students towards the end of their medical education. Based on our findings in the context of Wenger’s social learning theory and previous research, it seems necessary for medical students to gradually be given responsibility for patients and receive timely feedback that supports independent reasoning skills, as this may drive medical students forward in learning to become physicians.

While full membership in the nurses’ CoP is not a goal for medical students [41], we found that being legitimised as a member on a peripheral trajectory gave them a glimpse inside the nurses’ practice and insight into their CoP. Our findings showed that medical students learned to collaborate interprofessionally in mutual engagement with nursing students [41]. While the medical students performed the role and work tasks of a physician, they took advantage of the information that the nursing students provided about the patients. Collaboration with nursing students instead of nurses provided opportunities to exercise the physician’s role in an authentic manner and to practise professional communication and leadership skills. These findings are consistent with those of Andersen et al. [24] and indicate that when medical students work closely with nursing students, they experience how the collaboration between a physician and nurse can work. In addition, similar to the findings of Bondevik et al. [27], collaboration with nursing students provided insight into the role, work tasks, and thinking of the nursing students, which was considered valuable for future collaboration. Collaboration with nursing students can thus provide medical students with knowledge about nurses’ enterprise and repertoire [41]. With the increasing complexity of society and the patient population, it is necessary for medical students to learn to work in teams with other health professionals [1, 22]. Based on our findings, CoP theory, and previous research, we believe that municipal emergency care units can train medical students to collaborate interprofessionally and give them insight into the CoP of nurses.

The afternoon reflection meeting seemed to form a third CoP in which the medical students, together with the nursing students, nurses, and physicians, were legitimate participants [41]. We observed mutual engagement towards the joint enterprise of the meeting and noted instances in which the medical students used a shared repertoire to adapt to the nursing students [41]. Findings related to medical students’ learning and participation in afternoon reflection meetings suggested opportunities to develop in-depth knowledge about the topics discussed. As described by Gudmundsen et al. [30], setting aside time for professional reflections allowed the medical and nursing students, nurses, and physicians to expand on their knowledge and experiences. Consistent with Newbronner et al. [36], the medical students especially valued the physicians’ engagement when elaborating on medical topics. The medical students reported that they learned about the nurses’ role when the nursing students shared their perspectives and thinking. This finding coincides with the studies of Aase et al. [32] and van Lierop et al. [31], who found that meetings in which medical and nursing students participate can be a valuable arena for learning about other professions. Our findings, CoP theory and earlier research suggest that facilitating interprofessional reflections with nursing students, nurses, and physicians may provide medical students with in-depth knowledge about their own profession and the nursing profession.

### Strengths and limitations

The trustworthiness of the study was maintained by including the aspects of credibility, dependability, confirmability, and transferability in the methodological consideration process [58]. To strengthen the credibility, the medical students were chosen by purposive sampling based on predetermined criteria. Although the data are based on a predetermined sample of six participants, we cannot claim data saturation [49]. However, the interview transcripts provided rich data, and triangulating with the observational data contributed to strengthening the findings [49]. Thus, our findings suggest what learning opportunities medical students may encounter in municipal emergency care unit clerkships. The setting studied may not be automatically applicable in other countries, but the results can be transferred to similar contexts. While undertaking systematic observations, we recognised that our presence could distract medical students [48]. To minimise this effect, we adapted to the clinical environment by wearing medical uniforms supplied by the unit. When analysing the data, we did not consider the individual cognitive perspective but focused solely on the sociocultural perspective. Sfard [51] refers to these perspectives as the acquisition and participation metaphors, respectively. According to Sfard [51], one perspective does not exclude the other. While the individual cognitive perspective sheds light on how a person develops and constructs knowledge, the social learning perspective describes how learning occurs within social relations. However, we used both perspectives to inspire the question development for the interview guide.

## Conclusion

The findings of the present study suggest that this form of clerkship at municipal emergency care units may be a valuable learning arena for medical students to take on responsibility for emergency care patients and train in relevant work tasks by gradually participating in the physicians’ CoP. Interprofessional collaboration with nursing students on joint patients seems to help medical students learn the physician’s role in an authentic way, as well as give insight into the nurses’ CoP and how closely physicians partner with nurses in patient treatment. In addition, nursing students and nurses may be valuable assets for learning clinical procedures. The allocation of time for professional reflections with nursing students, nurses, and physicians seemed to create an interprofessional CoP that provided time and space for in-depth reflections about ethical and professional topics relevant to this setting. Based on our findings, we propose that municipal emergency care units can provide medical students with appropriate training in emergency medicine for patients in the primary health care system. Further research should focus on what nursing students learn and how they experience their participation in clerkships with medical students in similar settings.

## Supplementary Information

**Additional file 1.** Standards for Reporting Qualitative Research (SRQR).

## Data Availability

The dataset analysed is not publicly available due to a written agreement between the researchers and the study participants stating that no one but the authors of this article may read the transcripts, but anonymised data can be obtained from the corresponding author upon reasonable request.

## Abbreviations

CoP: Community of Practice
MS: Medical student
WHO: World Health Organisation
